# Comparison of cardiac function index derived from femoral and jugular indicator injection for transpulmonary thermodilution with the PiCCO-device: A prospective observational study

**DOI:** 10.1371/journal.pone.0200740

**Published:** 2018-07-31

**Authors:** Alexander Herner, Markus Heilmaier, Ulrich Mayr, Roland M. Schmid, Wolfgang Huber

**Affiliations:** Medizinische Klinik und Poliklinik II, Klinikum rechts der Isar der Technischen Universität München, Munich, Bavaria, Germany; Scuola Superiore Sant'Anna, ITALY

## Abstract

**Introduction:**

Cardiac function index (CFI) is a trans-pulmonary thermodilution (TPTD)-derived estimate of systolic function. CFI is defined as the ratio of cardiac output divided by global end-diastolic volume GEDV (CFI = CO/GEDV). Several studies demonstrated that the use of *femoral* venous access results in a marked overestimation of GEDV, while CFI is underestimated. One study suggested a correction formula for femoral venous access that markedly reduced the bias for GEDVI. Therefore, the last PiCCO-algorithm requires information about the CVC-position which suggests a correction of GEDV for *femoral* access. However, a recent study demonstrated inconsistencies of the last PiCCO algorithm using incorrected GEDV to calculate CFI despite obvious correction of GEDV. Nevertheless, this study was based on mathematical analyses of data displayed in a total of 15 patients equipped with only a *femoral*, but not with a *jugular* CVC. Therefore, this study compared CFI derived from the *femoral* indicator injection TPTD to data derived from *jugular* indicator injection in 28 patients with both a jugular and a femoral CVC.

**Methods:**

28 ICU-patients with PiCCO-monitoring were included. Each dataset consisted of three triplicate TPTDs using the *jugular* venous gold standard access and the *femoral* access *with* and *without* information about the femoral indicator injection to evaluate, if correction for femoral GEDV also pertains to CFI. (CFI_jug: *jugular* indicator injection; CFI_fem: *femoral* indicator injection; CFI_fem_cor: *femoral* indicator injection with correct information about CVC-position; CFI_fem_uncor: *femoral* indicator injection with *uncorrect* information about CVC-position; CFI_fem_uncor_form = CFI_fem_uncor * (GEDVI_fem_uncor/GEDVI_fem_cor)).

**Results:**

CFI_fem_uncor was significantly lower than CFI_jug (4.28±1.70 vs. 5.21±1.91 min^-1^; p<0.001). Similarly, CFI_fem_cor was significantly lower than CFI_jug (4.24±1.62 vs. 5.21±1.91 min^-1^; p<0.001). This is explained by the finding that CFI_fem_uncor was not different to CFI_fem_cor (4.28±1.70 vs. 4.24±1.62 min^-1^; p = 0.611). This suggests that correction for femoral CVC does not pertain to CFI. Calculative correction of CFI_fem_uncor by multiplying CFI_fem_uncor by the ratio GEDVI_fem_uncor/GEDVI_jug resulted in CFI_fem_uncor_form which was slightly, but significantly different from the gold standard CFI_jug (5.51±2.00 vs. 5.21±1.91 min^-1^; p = 0.024). The agreement of measurements classified in the same category of CFI (decreased (<4.5), normal (4.5–6.5) and increased (>6.5 min^-1^)) was high for CFI_jug and CFI_fem_uncor_form (identical categories in 26 of 28 comparisons; p = 0.49). By contrast, the agreement with CFI_jug was significantly lower for CFI_fem_cor (14 out of 28; p<0.001) and CFI_fem_uncor (15 out of 28; p<0.001).

**Conclusions:**

While the last PiCCO algorithm obviously corrects GEDVI for femoral indicator injection, this correction is not applied to CFI. Therefore, femoral TPTD indicator injection results in substantially lower values for CFI compared to TPTD using a jugular CVC. Necessarily, uncorrected CFI-values derived from femoral TPTD are misleading and have to be corrected.

## Introduction

Accurate haemodynamic monitoring is essential for the diagnosis and therapeutic management of critically ill patients with circulatory failure [1]. Different methods and techniques can be used to estimate the left ventricle (LV, Table 1) contractile fraction and the LV ejection function (LVEF). Doppler echocardiography is the gold standard imaging technique for measuring LVEF [2, 3], but repeated measurements are often not feasible due to the lack of experienced examiners on a 24/24h basis. Therefore, trans-pulmonary thermodilution (TPTD) has been suggested to assess cardiac systolic function [4–7]. In addition to stroke volume (SV) and cardiac output (CO) the preload parameter global end-diastolic volume (GEDV) and the marker of pulmonary edema extravascular lung water (EVLW) as well as a number of calculated ratios have been suggested to facilitate the interpretation of numerous parameters provided by TPTD and pulse contour analysis (PCA) [5, 8, 9]. Two of these parameters have been associated to cardiac contractility and with systolic function when compared to echocardiography or other gold-standard techniques (see overview of studies; Table 2) [4–7, 10–12].: Cardiac function index (CFI) and global ejection fraction (GEF). Both have been shown to accurately assess LVEF and its response to inotropic substances, volume depletion, resuscitation and impairment of contractility by verapamil [4, 10, 11, 13]. Previous studies also confirmed that a low CFI identified cardiac dysfunction in both acute heart failure and septic patients [4, 7, 10].

**Table 1:** Abbreviations and nomenclature of haemodynamic parameters.

LV	left ventricle
LVEF	LV ejection function
TPTD	trans-pulmonary thermodilution
SV	stroke volume
CO	cardiac output
CI	cardiac index
GEDV/GEDVI	global end-diastolic volume/ global end-diastolic volume index)
EVLW/EVLWI	extravascular lung water/ extravascular lung water index
PCA	pulse contour analysis
CFI	cardiac function index
CFI_jug	CFI determination based on jugular indicator injection
CFI_fem	CFI determination based on femoral indicator injection
CFI_fem_cor	CFI determination based on femoral indicator injection with the correct information about position of the CVC
CFI_fem_uncor	CFI determination based on femoral indicator injection without the correct information about position of the CVC
CFI_fem_uncor_form	
GEF	global ejection fraction
PVPI	pulmonary vascular permeability index
CVC	central venous catheter
LVFAC	left ventricular fractional area of change
ICU	intensive care unit
PAC	pulmonary artery catheter
PE	percentage error
CV	coefficients of variation
BW_ideal_	ideal bodyweight

**Table 2:** Overview of studies on cardiac function index (CFI).

Reference	Number of patients and measurements	Setting	Main result
Combes A. et al. [5]	n = 33 patients; comparison of CFI and GEF to left ventricular fractional area of change (LVFAC) derived from transesophageal echocardiography.	Intensive care unit (ICU)	CFI and GEF are closely associated to LVFAC except in patients with isolated right ventricular failure. ROC-AUCs were 0.92 for both CFI and GEF to predict a LVFAC≥40%.
Jabot J. et al. [4]	n = 39 patients; 48 measurements.	Medical ICU	Close association of CFI and GEF to left ventricular ejection fraction, respectively. Comparable changes of LVEF and CEF after dobutamine and 500mL saline (n = 24). ROC-AUC of 0.83 for CFI to detect a LVEF≤45%.
Ritter S. et al. [7]	n = 21 patients (12 with acute heart failure AHF, 9 with sepsis). PAC AND PiCCO. n = 84 measurements.	Medical ICU	Significant differences for CFI and GEF between patients with sepsis and AHF. Significant association of CFI and GEF with left ventricular stroke work index derived from PAC in patients with sepsis and AHF. Significant association of CFI with mixed venous oxygen saturation.
Trepte et al. [11]	16 pigs; 64 measurements before and after induction of hypovolaemia as well as before and after verapamil.	Animal study	CFI and GEF detect changes in preload independent cardiac contractility induced by verapamil. Both reflect changes of contractility induced by decrease of preload.
Belda et al. [6]	35 patients; 49 measurements. Comparison of CFI and GEF to left-ventricular ejection fraction (LVEF) derived from transthoracic echocardiography.	ICU-patients	Close association of CFI and GEF with LVEF r = 0.66 and r = 0.79; p<0.001). ROC-AUCs of 0.879 (GEF) and 0.805 (CFI) to predict LVEF <40%.
Mutoh et al. [12]	46 patients with subarachnoid hemorrhage (SAH). TPTD twice daily and transthoracic echocardiography once daily up to day 14.	Stroke ICU	A CFI value <4.2 min^−1^ had a sensitivity of 82% and specificity of 84% to predict LVEF <40%. The ROC-AUC was 0.85 which was superior to cardiac index.
Perny et al. [10]	n = 35 patients with cardiogenic shock. 90 measurements. Comparison to transthoracic echocardiography.	ICU-patients	CFI is significantly correlated to LVEF (p<0.001) in cardiogenic shock with the exception of patients with severe isolated right ventricular dysfunction. A CFI value <3.5 min^−1^ had a sensitivity of 81% and specificity of 63% to predict LVEF <35%. The ROC-AUC was 0.8.

Based on their mathematical derivation, CFI (CO/GEDV) and GEF (4*SV/GEDV) necessarily depend on an accurate determination of GEDV. However, several studies demonstrated that GEDV is markedly overestimated in case of using a femoral venous access for TPTD-indicator injection instead of a jugular or subclavian access [14–16]. Consequently, CFI is underestimated in case of femoral indicator injection [17]. One of these studies suggested a formula to correct GEDVI for femoral venous access that markedly reduced the bias for GEDVI in a small validation group [15]. Consequently, the last PiCCO-algorithm requires information about the central venous catheter (CVC)-position that results in correction of GEDVI if the information of a femoral access is given. However, at least two studies suggest inconsistencies of the last PiCCO-algorithm using uncorrected GEDV to calculate CFI and pulmonary vascular permeability index (PVPI) despite obvious correction of GEDVI in case of femoral indicator injection [17, 18]. Nevertheless, these studies were based on mathematical analyses of data displayed in a total of 15 patients equipped with only a femoral, but not with a jugular CVC.

Another recent study in patients equipped with both jugular and femoral CVCs demonstrated that the last PiCCO-algorithm corrects GEF, but not PVPI which resulted in a substantial underestimation of PVPI in case of femoral indicator injection [19]. However, in the study performed by Huber et al. the CFI was not analyzed [19].

Therefore, we have compared in the present study CFI values of 28 patients equipped with both jugular and femoral CVCs: Two triplicate measurements with femoral indicator injection with and without giving the information of femoral indicator injection were compared to the gold-standard of CFI derived from jugular indicator injection.

## Materials and methods

This prospective observational study was conducted in a ten-bed general ICU at a university hospital between October 6, 2016 and March 31, 2017. The study was approved by institutional review board approved (Ethikkommission; Fakultät für Medizin; Technische Universität München 3049/11s). Written informed consent was obtained by all patients (18 years of age or older) or their legal representatives. All patients had to be equipped with PiCCO device and with both jugular and femoral catheter. The indication for PiCCO monitoring was made independently from the study by the ICU physician in charge None of the patients had been included in one of the previous studies or databases comparing TPTD-parameters derived from jugular to femoral indicator injection [14, 15, 17–19]. After fulfilling above-mentioned criteria no patient was excluded.

The abbreviations and nomenclature of haemodynamic parameters used in this paper are summarized in Table 1.

28 datasets including triplicate TPTD with 15 ml cold saline solution were recorded in 28 patients equipped with both jugular and femoral CVC. The jugular venous access was used as the gold standard TPTD_jug. Furthermore, two triplicate TPTDs were performed via the femoral access *with* (TPTD_fem_cor) or *without* (TPTD_fem_uncor) the information about the *femoral* indicator injection. This was done to evaluate, if correction for femoral GEDV pertains to CFI_fem.

The three TPTDs were performed in a random order with the intention to avoid a systematic bias by repeated triplicate TPTDs with a total volume of 9*15 ml.

All measurements were performed in patients equipped either with conventional jugular and femoral CVC or conventional CVC and a dialysis catheter irrespective of the study. According to the local standard CVCs or dialysis catheters were inserted in different positions (one in the superior vena cava and the other one in the inferior vena cava).

A 5-lumen CVC (Multicath 5, Vygon; Aachen, Germany) with a maximum intravascular length of 20 cm and a diameter of 3.15 mm (9.5 French (Fr)) or a Gambro Gam Cath Dolphin dialysis-catheter (Gambro Gam Cath Dolphin; Gambro Hospal GmbH, Gröbenzell, Germany) was used for TPTD indicator injections. For femoral access dialysis catheters with a length of 250 mm and a diameter of 13 Fr were used. For jugular RRT access we used catheters with a length of 150–175 mm and a diameter of 13 F. The dialysis catheters were prefilled with ice cold saline immediately before the 1^st^ indicator injection, since the larger volume of the dialysis catheters (up to 1.6 ml) might result in a loss of indicator (1.6 ml of 15 ml, i.e. 11% of the indicator) and in a consecutive overestimation of volumetric parameters for the 1^st^ of TPTD-measurement. Femoral venous catheters were completely (total length of the vascular part) inserted under ultrasound guidance. The position of the tip was controlled (and corrected) according to X-ray for all jugular, but not for the femoral venous catheters.

TPTD was performed as previously described [15, 20, 21] using a 5 Fr thermistor-tipped arterial catheter (PV2015L20-A PiCCO catheter; Pulsion Medical Systems SE, Feldkirchen, Germany) with a length of 20 cm (5 Fr) placed in the femoral artery and a PulsioFlex or PiCCO-2-monitor (Pulsion Medical Systems SE, Feldkirchen Gemany) with the most recent algorithm requiring information about the venous catheter site (V3.1 algorithm).

### Statistical analyses

All data were controlled for input data error. Continuous variables are expressed as mean±standard deviation. Categorical variables are expressed as percentages. Wilcoxon-test for paired samples was used to compare continuous variables.

Bland-Altman analysis was used for the analysis of the agreement between CFI derived from jugular vs. femoral venous catheter sites for CFI and to compute the percentage error (PE).

The agreement of classification of CFI (decreased (<4.5 min^-1^), normal (4.5–6.5 min^-1^) and increased (>6.5 min^-1^)) derived from different measurements was primarily analyzed using Fisher´s exact test (“agreement yes or no”). Additionally, we calculated kappa-statistics and Kendall´s coefficient of correlation.

All statistical analyses were performed using the IBM SPSS Statistics software version 23 (SPSS Inc., Chicago, IL, USA).

#### Sample size calculation

The sample size calculation based on the finding of the previous study by Beitz et al. with CFI-values calculated for jugular indicator-injection of 5.1±1.8 min^-1^ and significantly lower values of 3.8±1.6 min^-1^ derived from uncorrected femoral indicator injection, using an online statistical power calculator (https://www.dssresearch.com/KnowledgeCenter/toolkitcalculators/statisticalpowercalculators.aspx) Sample sizes of n = 12 and n = 16 would provide statistical powers of 80% and 90% respectively [17]. However, the data by Beitz et al. were calculatorily derived from 95 datasets from only 10 patients [17]. A larger number of patients might have resulted in a stronger standard deviation of the CFI-value measured after femoral indicator injection. Therefore, we assumed the same mean values for CFI, but for power calculation a higher standard deviation of 2.1 min^-1^ instead of 1.6 min^-1^ in the study by Beitz et al.. This resulted in a sample size of n = 28 to provide a statistical power of 90% and an alpha-error of 5% (two-tailed test).

## Results

### Patients characteristics

Table 3 shows the patients characteristics.

**Table 3:** Patients characteristics.

Patients characteristics	
Sex (male : female; n (%))	14 : 14 (50%:50%)
Age (years±SD)	65±13
Height (cm±SD)	172±10
Weight (kg±SD)	85±25
Underlying disease (n (%))	
- Sepsis	8/28 (28.6%)
- ARDS	13/28 (46.4%)
- Cirrhosis/HRS	3/28 (10.7%)
- Cardiogenic shock	1/28 (3.6%)
- Severe pancreatitis	3/28 (10.7%)
SOFA (score±SD)	9.0±4.3
APACHE II (score±SD)	17±4
Tidal volume (ml/kg)	6.4±2.4
Measurements under vasopressors (norepinephrine, dobutamine and terlipressin)	22/28 (79%)
Measurements under mechanical ventilation	25/28 (89%)
Measurements under controlled ventilation	14/28 (50%)
Heart rhythm (sinus rhythm : atrial fibrillation)	25 : 3 (89% : 11%)
Measurements under sinus rhythm and controlled ventilation	14/28 (50%)
Ejection fraction (n (%)	
<30%	6/24 (25%)
30–44%	2/24 (8%)
45–54%	14/24 (58%)
≥55%	14/24 (58%)

### Comparison of CFI-measurements using different indicator injection sites (all measurements)

**Fig 1:**
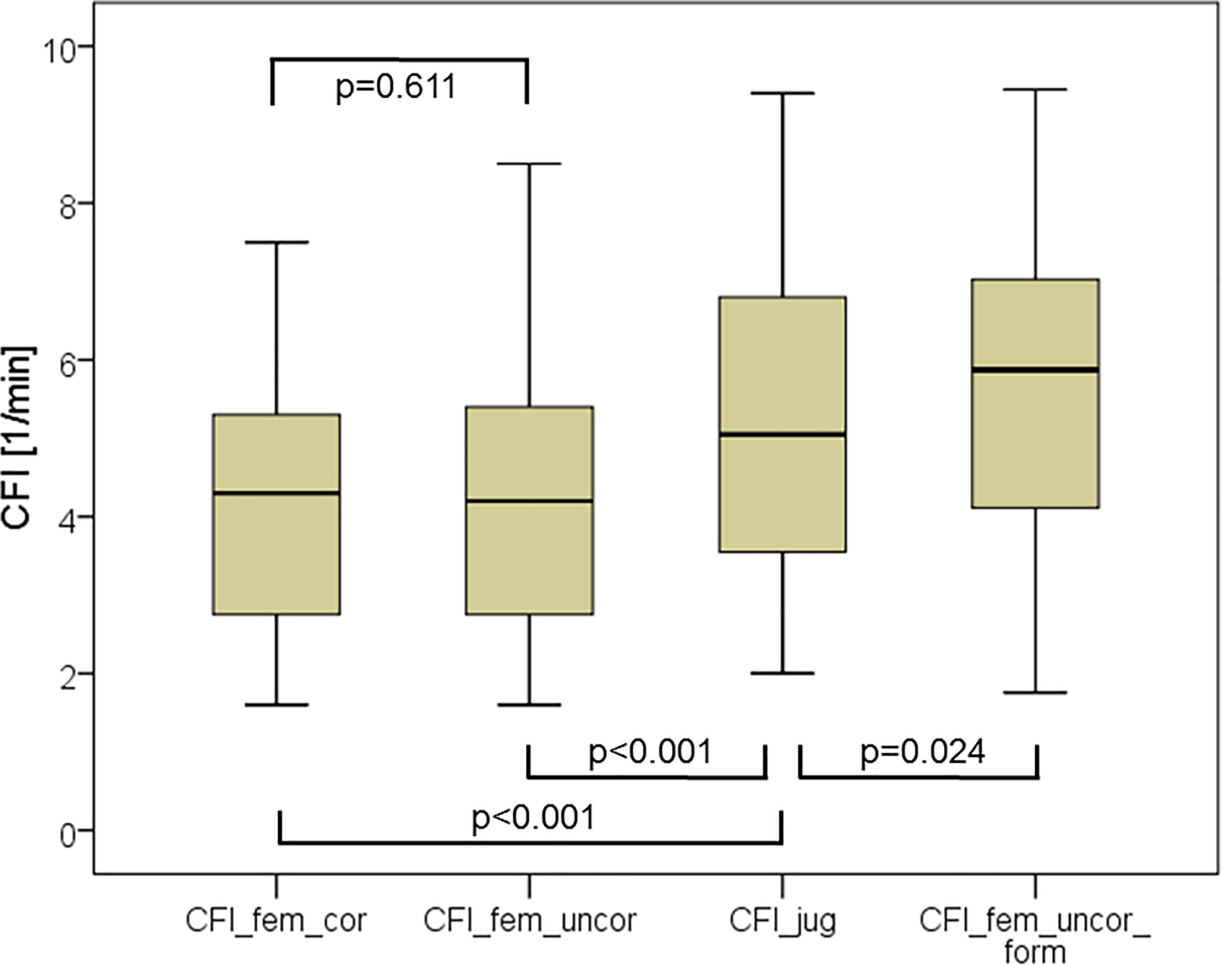
Boxplots plots comparing cardiac function index (CFI) derived from jugular indicator injection (CFI_jug), from femoral injection without activation of a potential correction by the device (CFI_fem_uncor), from femoral injection with activation of a potential correction by the device (CFI_fem_cor) and from femoral injection without activation of a potential correction by the device, but corrected by the previously suggested formula (CFI_fem_uncor_form). CFI_fem_uncor_form was corrected using the formula suggested for correction of femoral indicator injection derived GEDVI: GEDVI_corrected_ [ml/m^2^] = 0.539 * GEDVI_uncorrected_—15.17 + 24.49 * CI_uncorrected_ 2.311* BW_ideal._ CFI_fem_uncor_form was calculated by multiplying CFI_fem_uncor with the ratio GEDV_uncorrected_/GEDV_corrected_. GEDV(I): global end-diastolic volume (index).

**Fig 2:**
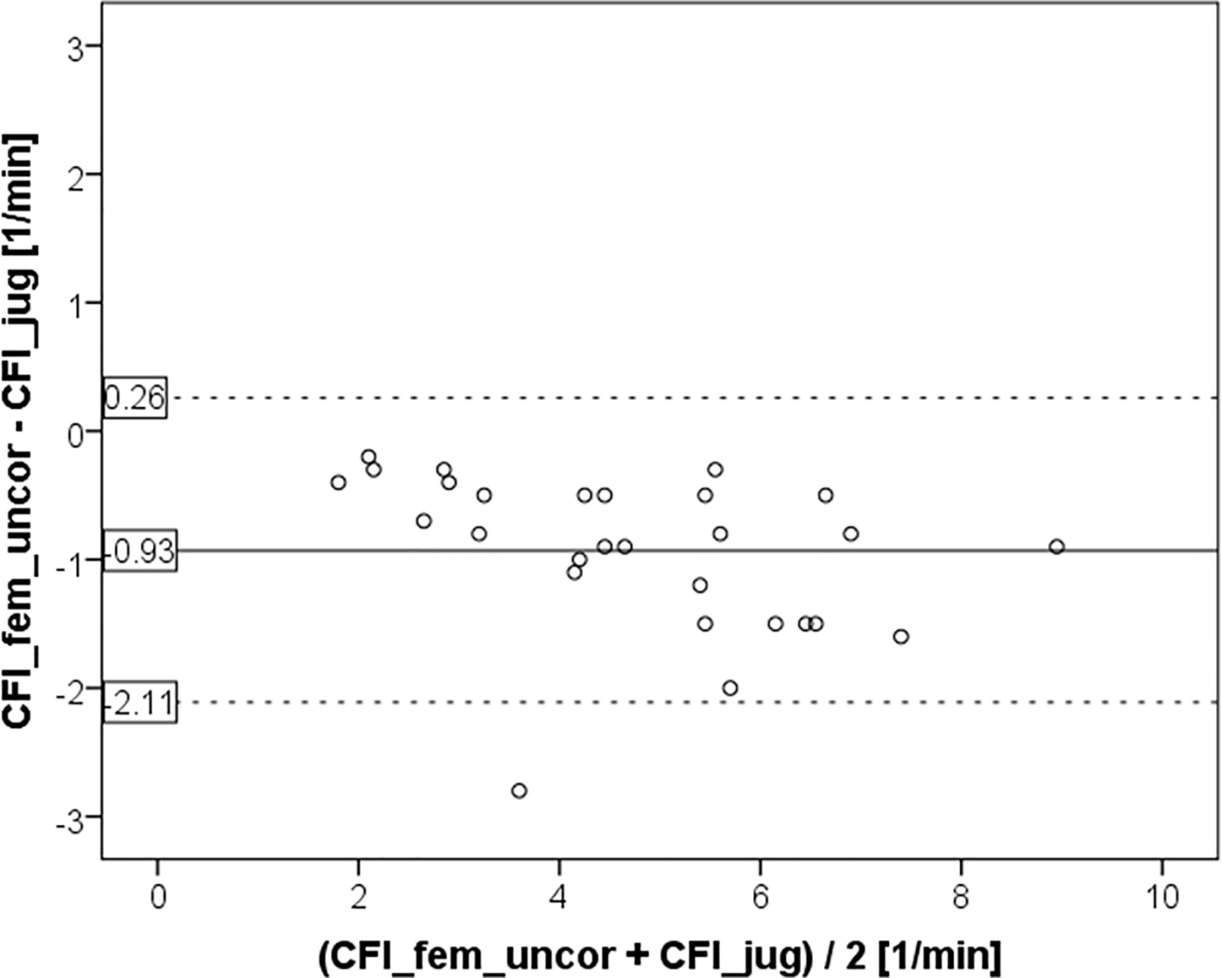
Bland Altman plot comparing cardiac function index CFI_fem_uncor derived from femoral injection without activation of a potential correction by the device to the gold standard measurement CFI_jug using a jugular CVC for indicator injection.

CFI_fem_uncor was significantly lower than CFI_jug (4.28±1.70 vs. 5.21±1.91 min^-1^; p<0.001; Fig 1 and Fig 2) with a bias of -0.93±0.60 and a percentage error of 25%. The coefficients of variation (CV) were in the same range for CFI_jug and CFI_fem_uncor (39.7% and 36.6%, respectively).

**Fig 3:**
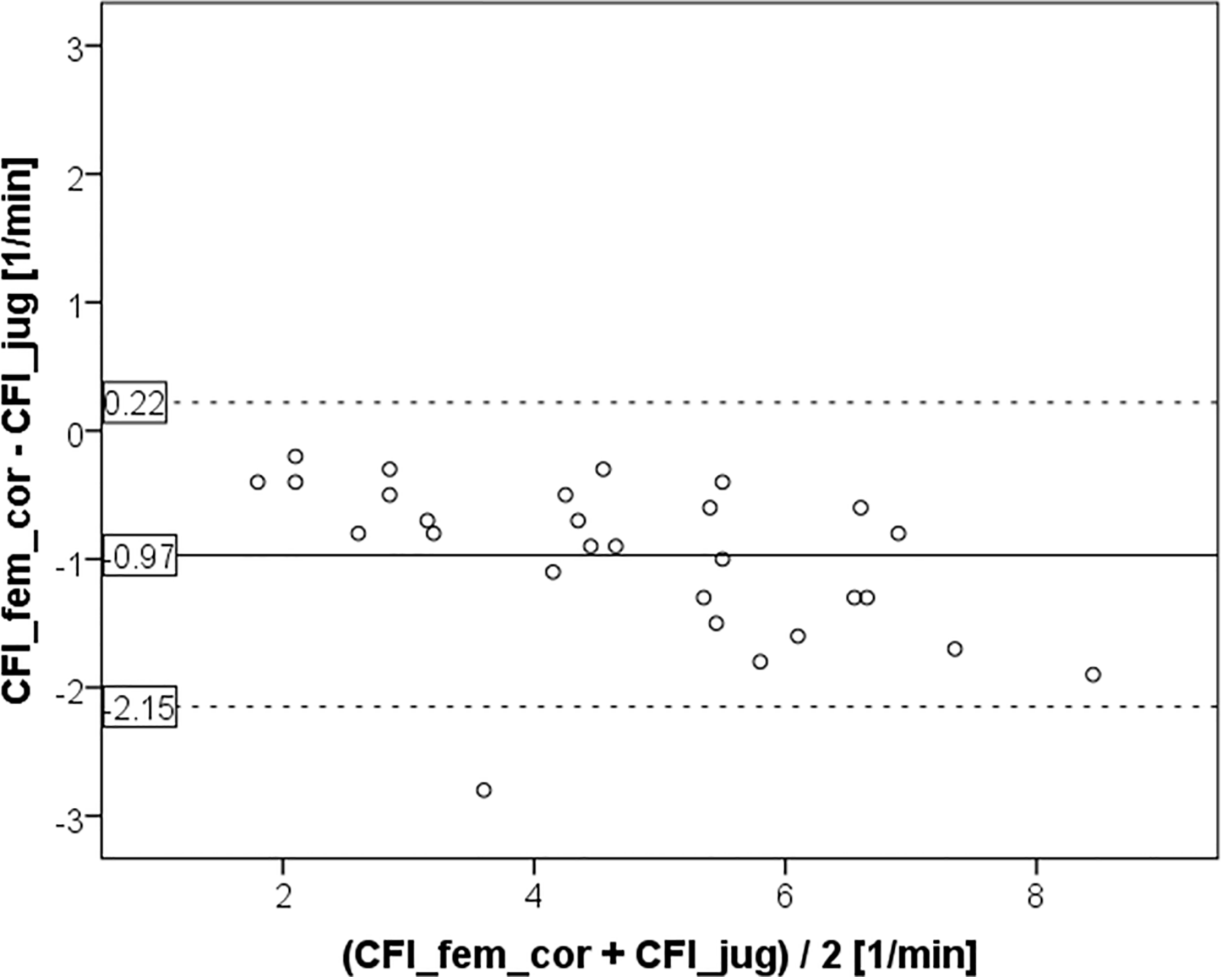
Bland Altman plot comparing cardiac function index CFI_fem_cor derived from femoral injection after activiation of a potential correction by the device to the gold standard measurement CFI_jug using a jugular CVC for indicator injection.

Similarly, CFI_fem_cor was significantly lower than CFI_jug (4.24±1.62 vs. 5.21±1.91 min^-1^; p<0.001; Fig 1 and Fig 3). This resulted in a bias of 0.97±0.60 and a percentage error of 25%. CV was comparable for CFI_jug and CFI_fem_cor (39.7% and 38.2%, respectively).

**Fig 4:**
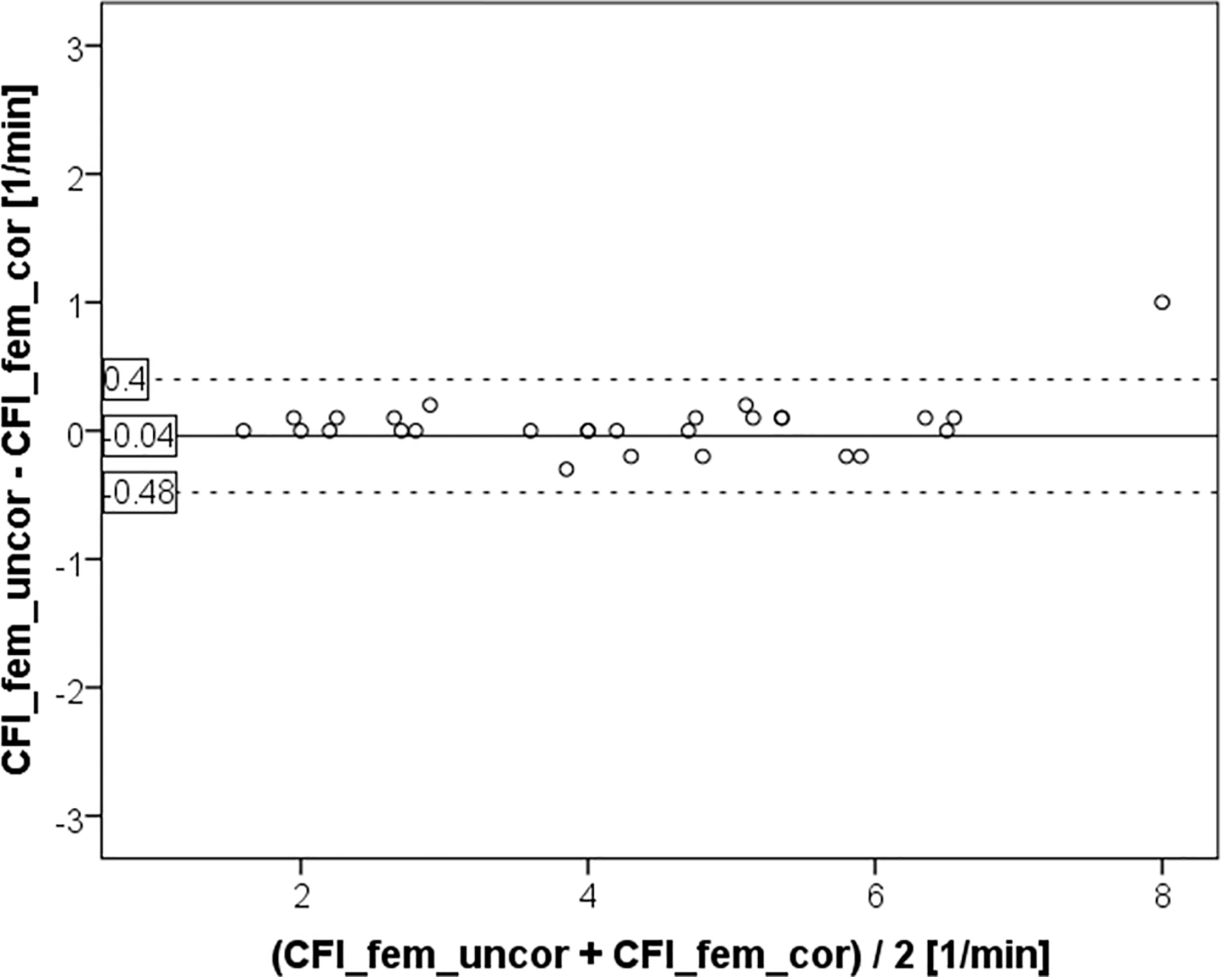
Bland Altman plot comparing cardiac function index CFI derived from femoral indicator injection with (CFI_fem_cor) and without (CFI_fem_uncor) activation of a potential correction by the device.

This is explained by the finding that CFI_fem_uncor was not different to CFI_fem_cor (4.28±1.70 vs. 4.24±1.62 min^-1^; p = 0.611; Fig 1 and Fig 4) with a bias of 0.04±0.22 min^-1^ and a PE of 10%.

The relation of CFI_jug/CFI_fem_uncor (5.21 min^-1^/4.28 min^-1^; 122%) was in the same range as the ratio of GEDVI_fem_uncor/GEDVI_fem_cor (1110 ml/m^2^/838 ml/m^2^; 132%).

Therefore, CFI_fem_form was calculated by correcting CFI_fem_uncor by multiplication of CFI_fem_uncor with the ratio GEDVI_fem_uncor/GEDVI_fem_cor using the recently suggested correction formula for GEDVI_fem based on uncorrected GEDVI and cardiac index (CI) derived from femoral indicator injection (GEDV_fem_uncor and CI_fem_uncor) as well as on ideal bodyweight (BW_ideal_) [15]:

$$GEDVI\_fem\_cor\;\left[ml/m^{2}\right]=0.539\;*GEDVI\_fem\_uncor‑15.17+24.49\;*CI\_fem\_uncor+2.311\;*BW_{ideal.}$$

Consequently, for ex-post-correction of CFI_fem_uncor we calculated CFI_fem_uncor_form by multiplying CFI_fem_uncor with the ratio GEDVI_fem_uncor/GEDVI_fem_cor:

CFI_fem_uncor_form=CFI_fem_uncor*(GEDVI_fem_uncor/GEDVI_fem_cor).

**Fig 5:**
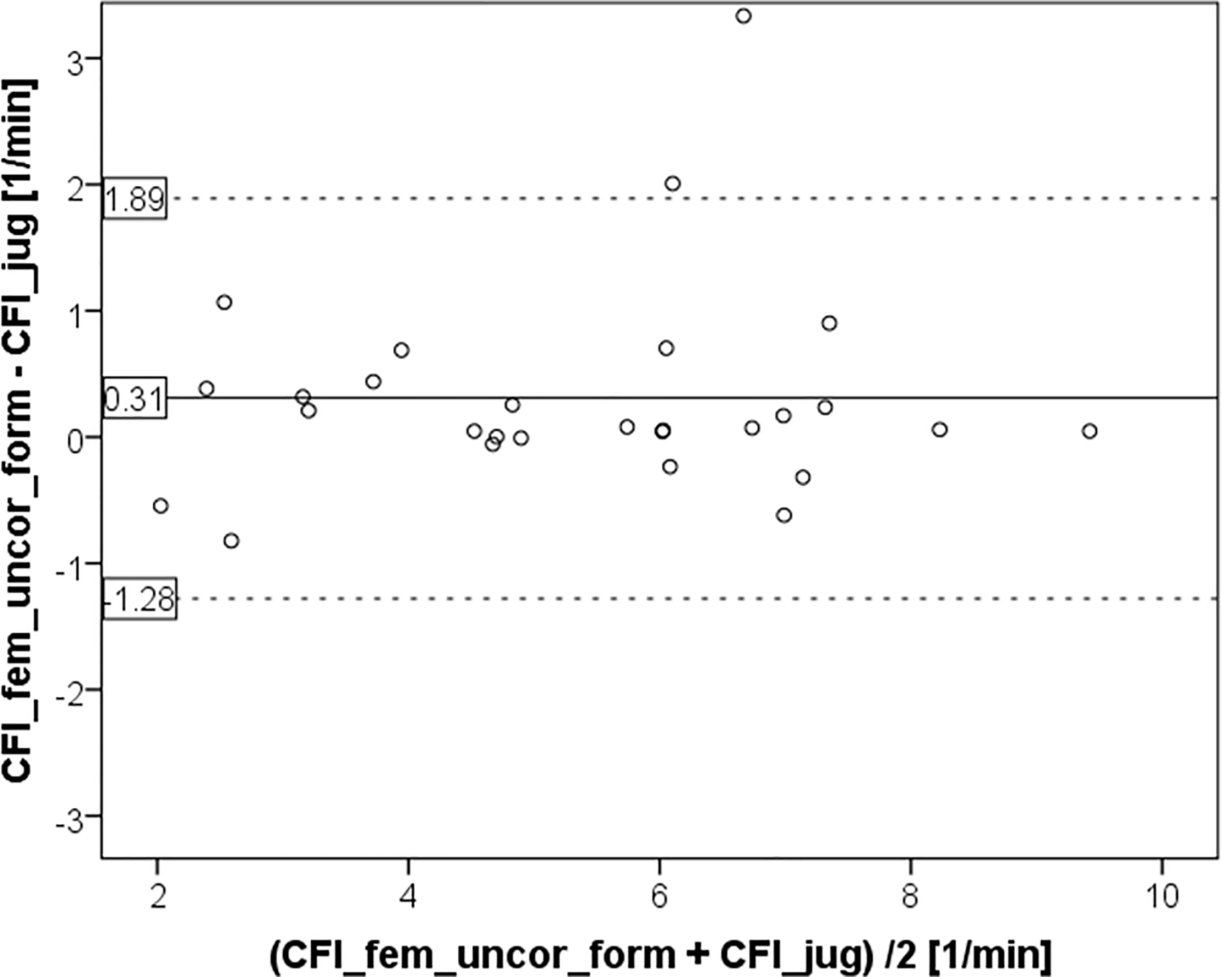
Bland Altman plot comparing cardiac function index CFI_jug derived from jugular indicator injection to cardiac function index CFI-fem_uncor_form which was derived from femoral indicator injection without activation of a potential correction by the device, but corrected with the formula suggested for correction of femoral indicator injection derived global end-diastolic volume index GEDVI: GEDVI_corrected_ [mL / m^2^] = 0.539 * GEDVI_uncorrected_—15.17 + 24.49 * CI_uncorrected_ 2.311* BW_ideal._ CFI_fem_uncor_form was calculated by multiplying CFI_fem_uncor with the ratio GEDV_uncorrected_/GEDV_corrected_. GEDV(I): global end-diastolic volume (index). CI: Cardiac index.

CFI_fem_uncor_form was slightly, but significantly different from the gold standard CFI_jug (5.51±2.00 vs. 5.21±1.91 min^-1^; p = 0.024; Fig 1 and Fig 5) with a bias of 0.30±0.81 min^-1^ and a percentage error of 29.6%. The CV-values were comparable for CFI_fem_uncor_form and CFI_jug (36.3% and 39.7%, respectively).

Despite a slightly significant difference between CFI_fem_uncor_form and CFI_jug with questionable clinical relevance, ex-post correction of CFI_uncor resulted in significantly lower amount of the bias |CFI_fem_uncor_form–CFI_jug| vs. |CFI_fem_uncor–CFI_jug| (0.49±0.71 vs. 0.93±0.60 min^-1^; p = 0.005).

To further evaluate the impact on the potential clinical decision process we compared the distribution of elevated, normal and decreased CFI-values (>6.5 min^-1^, 4.5–6.5 min^-1^, <4.5 min^-1)^ for the “gold-standard” CFI_jug vs. the classifications according to CFI_fem_cor, CFI_fem_uncor and CFI_fem_uncor_form, respectively (Table 4). The agreements of CFI_fem_uncor and CFI_fem_cor were 15 out of 28 (54%) and 14 out of 28 (50%), respectively. Both comparisons were significantly different to the gold standard of CFI_jug (p<0.001; Fisher´s exact test).

**Table 4:** Categories of cardiac function index (CFI) classified as decreased (CFI <4.5 min^-1^), normal (4.5≤ CFI ≤6.5 min^-1^) and increased (CFI >6.5 min^-1^).

		CFI__fem_cor_ [min^-1^]			CFI__fem_uncor_ [min^-1^]			CFI__fem_uncor_form_ [min^-1^]		
		<4.5	4.5≤ CFI ≤6.5	<4.5	<4.5	4.5≤ CFI ≤6.5	>6.5	<4.5	4.5≤ CFI ≤6.5	>6.5
**CFI**_**_jug**_ **[min**^**-1**^**]**	<4.5	8 (28.6%)	0 (0.0%)	0 (0.0%)	8 (28.6%)	0 (0.0%)	0 (0.0%)	8 (28.6%)	0 (0.0%)	0 (0.0%)
	4.5**≤** CFI **≤** 6.5	7 (25.0%)	5 (17.9%)	0 (0.0%)	7 (25.0%)	5 (17.9%)	0 (0.0%)	0 (0.0%)	10 (35.7%)	2 (7.1%)
	>6.5	0 (0.0%)	7 (25.0%)	1 (3.6%)	0 (0.0%)	6 (21.4%)	2 (7.1%)	0 (0.0%)	0 (0.0%)	8 (28.6%)

The agreement of CFI_fem_uncor_form was 26/28 (92.6%), which was not significant different to CFI_jug (p = 0.49; Fisher´ s exact test), but significantly higher compared to CFI_fem_uncor (p = 0.0019) and CFI_fem_cor (p<0.001).

Furthermore, kappa-statistics and Kendall´s coefficient tau-b confirm a markedly better agreement of CFI_fem_uncor_form with CFI_jug (kappa = 0.892; p<0.001; Kendall´s coefficient tau-b = 0.930; p<0.001) compared to CFI_fem_uncor (kappa = 0.295; p = 0.0018; Kendall´s coefficient tau-b = 0.721; p<0.001) and CFI_fem_cor (kappa = 0.234; p = 0.057; Kendall´s coefficient of correlation = 0.717; p<0.001).

## Discussion

TPTD-derived CFI is a bedside surrogate of LV systolic function. CFI is strongly associated with echocardiography-derived LVEF and facilitates guidance of inotropic therapy and fluid management. Repeated CFI measurement is readily available and independent of the examiner. Changes in CFI over time provide dynamic information that might be superior to single measurements, particularly when interpreted in the light of a clinical situation. According studies with their findings are summarised in Table 2.

However, the validity of CFI calculation relies on the accurate determination of CO and GEDV. Several recent studies suggest marked overestimation of GEDV/GEDVI and CFI in case of using a *femoral* CVC for indicator injection, compared to the gold standard of *jugular or subclavian* injection. Interestingly, a similar phenomenon was found in case of misplacement of subclavian central venous catheter tip into the jugular vein [22].

One study suggested a correction formula for GEDVI derived from femoral indicator injection. This correction is based on GEDVI and CI obtained from femoral access and on ideal bodyweight [15]. Several studies suggest that a similar formula has been integrated to the last PiCCO-2-algorithm [17, 18]. However, based on mathematical analyses of the data displayed for CFI and GEF, one recent study suggested that CFI obviously is not corrected for femoral injection [17]. Since analyses were performed in small cohorts with only femoral CVCs, the final proof of these results in patients equipped with both jugular and femoral catheters remained to be demonstrated.

This study demonstrates that the correction formula for femoral venous access is not applied to correct CFI. The resulting underestimation of this value would have had a consequence for around 50% of our patients as demonstrated by the wrong classification of CFI in 14 out of 28 measurements. Therefore, measurement of CFI in patients with femoral venous access for indicator injection at present is misleading and has to be replaced by echocardiography as long as the correction formula is not implemented in the TPTD-algorithm. This problem might also apply to the other commercially available TPTD-device EV-1000 (Edwards Lifesciences, Irvine, USA), since at least one study suggests that this device does not correct GEDVI, PVPI and GEF for femoral indicator injection [14].

Unless an appropriate correction is implemented in the PiCCO and the EV-1000, in patients with *femoral* central venous access echocardiography should be performed to assess left ventricular contractility. Irrespective of the central venous access, echocardiography enables to exclude isolated right heart failure which might impede the use of CFI and GEF also in case of a jugular or subclavian central venous access [10].

However, repeated echocardiography is time consuming and requires the continuous availability of experienced investigators. On the other hand, TPTD is straightforward and reliable even when performed by different investigators. Furthermore, it provides additional extra-cardiac parameters such as EVLWI and calibrates continuous measurement of CI, GEF and CFI.

### Practical implication

Although a correction formula for femoral venous access markedly reducing the bias for GEDVI has been published 7 years ago, and despite several studies gave hints for inconsistencies of the correction of GEDVI, PVPI, GEG and CFI, our data demonstrate that the most recent algorithm of the PICCO still does not apply this correction to CFI. Therefore, in patients with *femoral* venous access CFI has to be classified as misleading and may result in wrong therapeutic interventions due to substantial underestimation and wrong categorization of CFI.

From a practical viewpoint, there are two options to overcome this dilemma in addition to the use of echocardiography:

1. As demonstrated by this study, ex post correction by the previously suggested formula to correct GEDVI appropriately corrects CFI_fem with acceptable bias, percentage error and categorization according to clinical thresholds.
2. Since this mathematical correction maybe cumbersome in clinical routine, GEF can be used instead of CFI. At least two studies suggest that GEF is appropriately corrected for femoral indicator injection by the most recent PiCCO algorithm [18, 19].

### Limitations of the study

This study included a small number of patients and has been conducted as a single-centre study. Furthermore, all measurements were performed in critically ill patients and not in healthy persons.

### Conclusion

While the last PiCCO algorithm obviously corrects GEDVI for femoral indicator injection, this correction is not applied to CFI. Therefore, *femoral TPTD* indicator injection results in substantially lower values for CFI compared to TPTD using a jugular CVC. Necessarily, uncorrected CFI-values derived from femoral TPTD are misleading and have to be corrected.
